# Feasibility of an At-Home Experimental Circadian Misalignment Induction for Adolescents

**DOI:** 10.3390/clockssleep7010004

**Published:** 2025-01-28

**Authors:** Dean W. Beebe, Andrea L. Fidler, Laura McLaughlin, Sabrina Grove, Stephanie J. Crowley

**Affiliations:** 1 Division of Behavioral Medicine and Clinical Psychology, Cincinnati Children’s Hospital Medical Center, Cincinnati, OH 45229, USA; laura.mclaughlin@cchmc.org (L.M.); sabrina.grove@cchmc.org (S.G.); 2 Department of Pediatrics, University of Cincinnati College of Medicine, Cincinnati, OH 45223, USA; 3 Department of Child & Adolescent Psychiatry and Behavioral Sciences, Children’s Hospital of Philadelphia, Philadelphia, PA 19104, USA; 4 Medical Sciences Program, University of Cincinnati College of Medicine, Cincinnati, OH 45267, USA; 5 Department of Psychiatry and Behavioral Sciences, Rush University System for Health, Chicago, IL 60612, USA; stephanie_crowley_mcwilliam@rush.edu

**Keywords:** sleep manipulation, circadian phase, adolescence

## Abstract

Despite extensive research on the effects of sleep restriction on adolescent health, the field lacks experimental methods to study the health effects of mistimed sleep, which is also common among adolescents. This paper describes a novel 3-week experimental protocol that was designed to compare sleep restriction, like what many adolescents experience on school nights, against sleep that meets the recommended duration but is timed to be relatively aligned or misaligned with their circadian phase. Healthy 14–18-year-olds, classified as early (“Lark”) and late (“Owl”) chronotypes, entered a six-night chronotype-aligned stabilization condition, followed by five nights of sleep restriction, a return to the stabilization schedule, and five nights of healthy sleep duration (HS). During HS, participants were randomly assigned to early-to-bed versus late-to-rise arms, intended to align with or misalign with their circadian phase. Actigraphy monitored sleep, and weekly dim-light melatonin onset (DLMO) assessed circadian phase. Analyses confirmed that the protocol met five key validation metrics related to differential attrition, sleep timing, circadian phase, and experimental induction of HS that is timed to be relatively aligned vs. misaligned with circadian phase. This protocol appears useful for future research into how misaligned sleep patterns, which occur regularly for many adolescents, may impact health.

## 1. Introduction

Adolescence has been characterized as “the perfect storm” of restricted and mistimed sleep [1,2]. There has been extensive research on the impacts of sleep *restriction* on the health and well-being of adolescents, but little research has examined the potential importance of sleep *timing* in that age range. On a basic level, the field lacks validated experimental protocols that systematically vary both sleep duration and sleep timing for adolescents; lacking those, we cannot test cause–effect relationships. To fill that foundational gap, this paper describes a novel experimental protocol that was designed to compare sleep restriction, like what many adolescents experience on school nights, against sleep that meets the recommended duration but is timed to be relatively aligned or misaligned with an adolescent’s circadian phase (see Table 1 for a glossary of key terms). Beyond simply describing the protocol, this paper empirically tests the protocol’s success across five validation metrics and outlines the strengths and challenges of using the protocol in future research with health outcome variables.

The need to experimentally study the impacts of adolescents’ sleep timing is rooted in well-documented developmental shifts in sleep physiology alongside psychosocial pressures that limit sleep opportunities [1,2]. Physiologically, both elements of the two-factor model of sleep regulation change during adolescence: the homeostatic sleep drive builds “sleep pressure” more slowly during wakefulness, and intrinsic circadian rhythms shift later relative to external time [1,2]. The former is permissive of staying up later in the evening, and the latter promotes it. As a result, sleep–wake patterns normatively shift later [1,3].

Despite the general trend toward later sleep–wake timing, tremendous individual differences remain: the variation in sleep–wake timing is as wide during adolescence as any other stage of life [1,3]. Relative to their peers, some adolescents are “morning larks” (or simply “Larks”) who prefer earlier bedtimes and wake times, while others are “night owls” (or “Owls”) who favor later schedules. In contrast to this variability, one relative constant in the US and many other developed countries is that schools for adolescents start as early as or earlier than those for younger children [4,5,6,7,8]. This causes many adolescents, especially Owls, to sleep far less than the recommended 8–10 h on school nights [5,6,8].

Considerable attention has been given to the consequences of short sleep during adolescence. Large-scale correlational studies converge with experimental research to confirm that chronic sleep restriction causally contributes to sleepiness, inattention, impaired learning, negative mood, poor affect regulation, reduced driving skills, increased somatic symptoms, and lower dietary quality [9,10,11,12,13,14,15,16,17,18,19,20,21,22,23]. This has led to efforts to alleviate sleep restriction. While advocacy for later school start times has had some impact [6], the practical reality for many adolescents is that—because they must awaken early for school—the only way to achieve adequate sleep is to go to bed early.

Compared to Larks, adolescent Owls find it more challenging to adopt early-to-bed efforts to sleep more [24]. There is also evidence that, even when Owls do fall asleep early, they may not reap the same benefits. In a study by Beebe and colleagues, adolescents’ dietary intakes were compared between a five-night late-to-bed sleep restriction condition and a five-night early-to-bed healthy sleep condition, with chronotype estimated from pre-randomization sleep–wake patterns [15]. Compared to short sleep, the healthy sleep condition increased both Larks’ and Owls’ sleep (~2.5 h/night on average), but that seemed to benefit only the Larks on the main outcome measured. Caloric intake was significantly less for Larks when they were well rested than when they were sleep-restricted. In contrast, Owls’ intake was similar under both sleep conditions and, overall, was as high as the Larks’ intake when they had inadequate sleep [15].

Such findings could be interpreted as evidence that being an Owl is unhealthy, independent of sleep duration, but there is an alternative hypothesis: the early-to-bed sleep extension condition may have been misaligned with Owls’ circadian phase. A person’s chronotype is the behavioral correlate of their circadian phase; Owls have later circadian phases than Larks [25], so an early-to-bed schedule is temporally misaligned with an Owl’s phase. This misalignment hypothesis is consistent with adult correlational data that link cardiometabolic morbidity, inattention, and poor decision-making to rotating shiftwork, in which there is a marked misalignment between circadian phase and sleep–wake schedules [26,27,28,29,30,31,32], as well as to more modest weeknight-to-weekend shifts in sleep schedules (which suggest recurrent misalignment) [33,34,35,36]. Such weeknight-to-weekend schedule shifts are also associated with poorer cognition in elementary students with ADHD [37] and altered reward processing in typically developing adolescents [38].

However, correlational data are often subject to hidden confounders. Distinguishing the impact of sleep duration from sleep timing is particularly challenging in correlational studies, as adolescent Owls often sleep the least on school nights and have the most variable sleep schedules [39]. To our knowledge, no published study has tested the misalignment hypothesis for adolescent Larks, as this would require a later sleep schedule than schools typically allow. Experimental research is needed that induces aligned versus misaligned sleep patterns across chronotypes. However, at present, the field lacks well-validated protocols for conducting such research.

**Figure 1:**
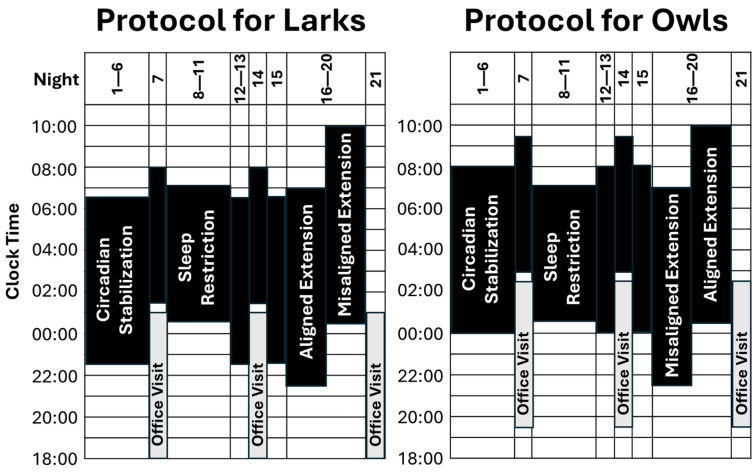
Overview of research design. Periods of sleep opportunity are denoted in black, with bedtime at the bottom of each bar and rise time at the top. As detailed in the text, there are three conditions of interest: circadian stabilization (SBN; nights 1–6; 8 h in bed, timed to fit Lark vs. Owl chronotype), sleep restriction (SR; nights 7–11; 6½ h in bed, timed between chronotypes), and sleep extension (EXT; nights 16–20; 9½ h in bed, randomized to early-to-bed vs. late-to-rise arms). Although not a focus of analyses, nights 12–15 acted as a 4-night washout between SR and EXT, during which participants returned to chronotype-specific sleep schedules. Sleep was monitored via actigraphy throughout the study, and circadian phase (DLMO) was measured at weekly office visits.

To help fill that gap, here, we detail an experimental protocol designed to test whether having a healthy sleep duration is uniquely impactful when it is aligned with adolescents’ circadian phase. As detailed in Section 4 (below) and summarized in Figure 1, this protocol combines intentional sample selection and a six-night chronotype-aligned circadian stabilization (SBN) condition to establish distinct chronotype groups (Larks and Owls) that differ in both sleep/wake schedules and circadian phase. Both groups then complete a five-night sleep restriction (SR) condition, with sleep opportunity limited to 6½ h per night. After returning for four nights to chronotype-specific schedules, participants close the study with a five-night sleep extension (EXT) condition of 9½ h/night sleep opportunity, randomly assigned to “Early-To-Bed” versus “Late-to-Rise” arms. Given the distinct Lark and Owl chronotypes, this random assignment during EXT is intended to experimentally induce sleep timing that is either aligned or misaligned relative to circadian phase. Throughout the study, objective actigraphy assesses sleep duration (onset to offset), timing (middle of the sleep period or “midsleep”), and quality (percent of sleep period spent sleeping; “efficiency”). Circadian timing (dim-light melatonin onset; DLMO) is assessed at weekly office visits.

We are conducting a large study using this sleep protocol to test whether aligned versus misaligned sleep schedules impact key outcomes, including dietary intake and mood, in adolescents aged 14–18 years. Here, we leverage preliminary data to assess whether the protocol is successful in systematically varying sleep duration and timing relative to adolescents’ circadian phase. We set the following validation metrics as evidence of success:

1. Is attrition unbiased? If the protocol results in differential loss of participants with respect to chronotype, sex, age, race/ethnicity, or random assignment, this could complicate its future applications. We predicted that retention would be unbiased on these variables.
2. Does the protocol induce intended changes in sleep duration that are similar across chronotypes and randomization? If sleep duration differed across these variables, this would suggest potential confounders that would need to be addressed in future research. We predicted that the protocol would induce intended sleep durations within each condition that are similar across chronotypes and randomization.
3. Does the protocol identify behaviorally and physiologically different Lark and Owl groups? This is important if we hope to induce clear aligned versus misaligned HS conditions by experimentally varying sleep timing. We predicted that Lark and Owl groups would differ in sleep timing and DLMO.
4. Does the protocol’s random assignment result in expected changes in sleep timing during HS? This is critical to drawing causal inferences based on that random assignment. We predicted that there would be experimentally induced differences in sleep timing post-randomization that are not evident pre-randomization.
5. Beyond the above points, does the random assignment result in relative alignment versus misalignment of participants’ sleep timing with their circadian phase? Demonstrating this would allow for confident use of the protocol with health-related outcome measures. We predicted post-randomization induction of sleep schedules that are misaligned versus aligned with circadian phase, as evidenced in differential associations between DLMO and sleep timing across randomization arms, as well as higher scores on a misalignment index.

## 2. Results

### 2.1. Sample Description

Of 81 enrolled participants, 13 were lost prior to randomization, primarily due to extreme non-adherence to initial study instructions or family schedule conflicts. Of the 68 randomized, 9 were lost due to family schedule conflicts or participant disinterest. Table 1 provides descriptive characteristics of the 81 initially enrolled and 59 in the final sample. There were no missing demographic data. Of adolescents with complete actigraphy data, three were missing DLMO for a visit due to participant illness or unexpected family circumstances. Of those with complete DLMO, three were missing actigraphy data for a condition due to device failure. Analyses reported below are based on cases with complete data for each measure.

### 2.2. Attrition Appeared Unbiased; It Did Not Systematically Vary Across Demographics, Chronotype, or Randomization (Validation Metric 1)

As shown in Table 2, there was no evidence of differential attrition across participant age, sex, race/ethnicity, or chronotype (*p* > 0.10). As intended, roughly half of those completing the study were randomized to each of the two arms of the EXT condition. This resulted in 46% ending the study in an EXT arm aligned with their chronotype and 54% in a misaligned arm.

### 2.3. The Protocol Induced Intended Changes in Sleep Duration That Were Similar Across Chronotypes and Randomization (Metric 2)

Table 3 summarizes findings from repeated-measures linear models predicting each primary and secondary outcome, listing effect sizes (partial eta-squared) for the main effects of condition (SBN, SR, EXT), chronotype, randomized EXT arm (early-to-bed vs. late-to-rise), and interactions involving condition. For sleep duration, there was a significant main effect only of condition, F (2, 51) = 198.0, *p* < 0.001. By design, sleep period duration was shortest during SR (6:25 ± 0:36), followed by SBN (7:42 ± 0:27) and then EXT (08:30 ± 0:43), with *p* < 0.001 for pairwise comparisons. Over three-fourths of the participants averaged > 8 h of nightly sleep during EXT, but none did so during SR. Neither chronotype nor random assignment arm significantly moderated these findings, nor did they show main effects.

Although we did not have specific hypotheses around sleep quality, sleep efficiency also showed only a main effect of condition, F (2, 51) = 7.6, *p* = 0.001; conditions with shorter sleep opportunities showed modestly higher sleep efficiency (SR = 90%; SBN = 88%; EXT = 87%). As an exploratory check on whether alignment vs. misalignment might impact sleep quality, we re-ran analyses, substituting that variable for the early versus late randomization arm. Again, only the condition variable showed a main effect, with no main or interaction effects involving the alignment/misalignment variable (*p* > 0.10).

### 2.4. The Protocol Resulted in Lark and Owl Groups with Different Sleep Timing and Circadian Phase (Metric 3)

As shown in Table 3, there was a significant main effect of chronotype on DLMO, F (1, 52) = 13.6, *p* = 0.001, with no significant main or interaction effects involving condition or randomization. Larks had earlier DLMOs than Owls immediately following SBN (21:38 ± 01:22 vs. 23:13 ± 01:20) and just prior to their entry into the EXT condition (21:20 ± 01:06 vs. 22:39 ± 01:06), *p* < 0.001. There also appeared to be an interaction between condition and randomization arm on DLMO, F (1, 52) = 10.7, *p* < 0.001. However, follow-up tests revealed no impact of randomization within any condition (*p* > 0.05).

### 2.5. The Protocol’s Random Assignment Resulted in Expected Changes in Sleep Timing During HS, but Not Pre-Randomization (Metric 4)

There were main effects of chronotype and randomization on midsleep, but these were each significantly moderated by condition, F (2, 51) > 67.7, *p* < 0.001 (Table 3). Given the significant interactions, follow-up tests examined the main effects of chronotype and randomization within each condition. As designed, Larks’ midsleep averaged ~1½ h earlier than that of Owls during the initial SBN condition (03:16 ± 0:36 vs. 05:38 ± 0:49, *p* < 0.001). Although the gap was smaller (~½ hour), Larks also had earlier midsleep during SR (03:52 ± 0:18 vs. 4:19 ± 0:36, *p* = 0.001). During the EXT condition, when sleep schedules were randomly assigned, there was no significant difference in midsleep across chronotypes (*p* > 0.10). Instead, during the EXT condition, adolescents randomized to the early-to-bed arm had significantly earlier midsleep than those in the late-to-rise condition (02:50 ± 0:31 vs. 05:14 ± 0:28, *p* < 0.001), an effect that was not evident between these groups before randomization (*p* > 0.10).

### 2.6. The Random Assignment Resulted in HS That Was Relatively Aligned Versus Misaligned with Participants’ Circadian Phase (Metric 5)

**Figure 2:**
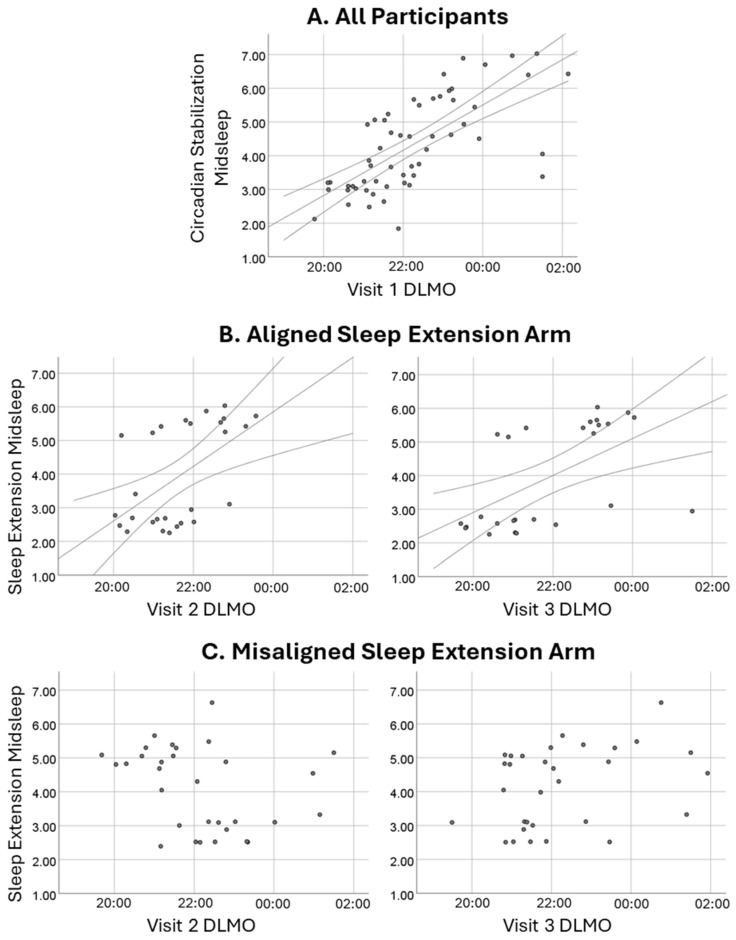
Midsleep–DLMO associations. Row (**A**) depicts the strong association between midsleep during the initial circadian stabilization condition and subsequent (Visit 1) DLMO in the full sample. Row (**B**) shows continued significant associations between sleep extension midsleep and DLMO at the visits just before (**left**) and after (**right**) for adolescents randomly assigned to the aligned extension arm. Row (**C**) shows weak, non-significant associations between sleep extension midsleep and DLMO at the visits just before (**left**) and after (**right**) for those randomized to the misaligned extension arm.

**Figure 3:**
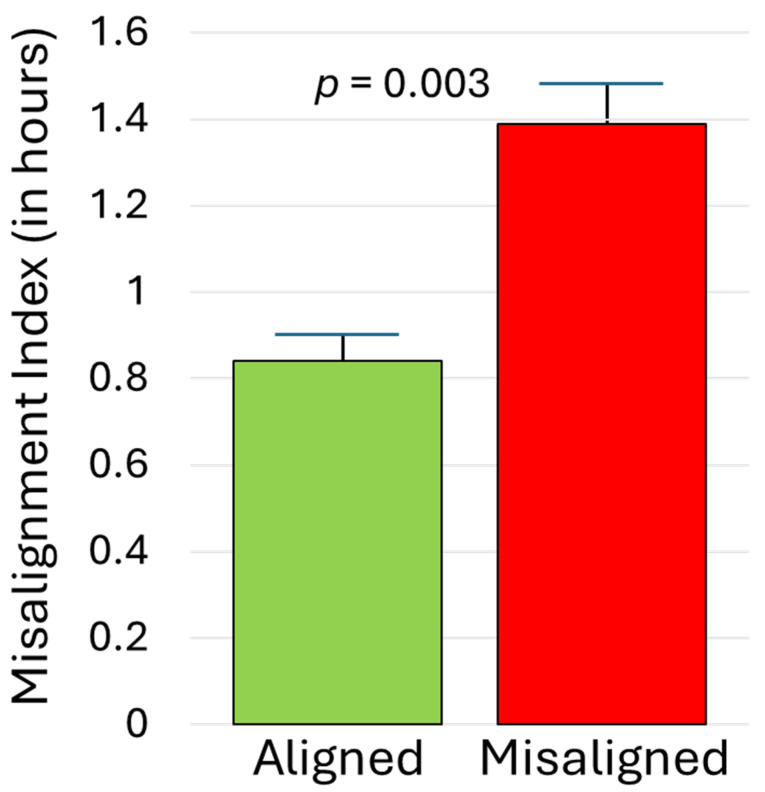
Misalignment index for adolescents randomized to aligned versus misaligned sleep extension. Higher scores reflect a circadian phase relationship (time interval from DLMO to actigraphic midsleep time) during the extension condition that deviates more from the phase relationship observed during the initial phase stabilization condition. Error bars represent standard errors of the mean.

As illustrated in Figure 2, midsleep during the initial SBN condition correlated strongly with subsequent DLMO, r_s_ = 0.74, *p* < 0.001. For those randomly assigned to the aligned EXT arm, midsleep during EXT was similarly significantly associated with DLMO obtained before (r_s_ = 0.55) and after (r_s_ = 0.70) the condition, *p* < 0.003. In contrast, among those randomized to the misaligned EXT arm, sleep timing and DLMO were effectively decoupled; midsleep during EXT had weak and non-significant associations with DLMO before (r_s_ = −0.34) and after (r_s_ = 0.34) the condition, *p* > 0.05. As detailed in the Methods, a misalignment index compared participants’ phase angle during EXT to that during SBN. As expected, that index was significantly higher for those assigned to the misaligned EXT arm than the aligned EXT arm (1.39 ± 0.82 versus 0.84 ± 0.40), *p* = 0.003. This effect was large (partial eta-squared = 0.158; see Figure 3) and was not moderated by chronotype or condition, *p* > 0.10.

## 3. Discussion

### 3.1. Main Findings

This experimental protocol was designed to compare short sleep, like what many adolescents experience on school nights, against sleep that meets the recommended duration but is timed to be relatively aligned or misaligned with an adolescent’s circadian phase. If successful, this protocol could allow for tests of the causal impact of such sleep schedules on key health variables, such as dietary intake, mood, and behavior, in this vulnerable population. As an initial step, this paper assessed whether the protocol met five key validation metrics.

Overall, the findings were very encouraging. First, while there was attrition across the study, it did not appear to be systematic by age, sex, race/ethnicity, or chronotype, and there was roughly equal representation of randomized arms in the final sample. This is important because differential attrition could complicate the interpretation of experimental effects; the present findings are reassuring.

**Figure 4:**
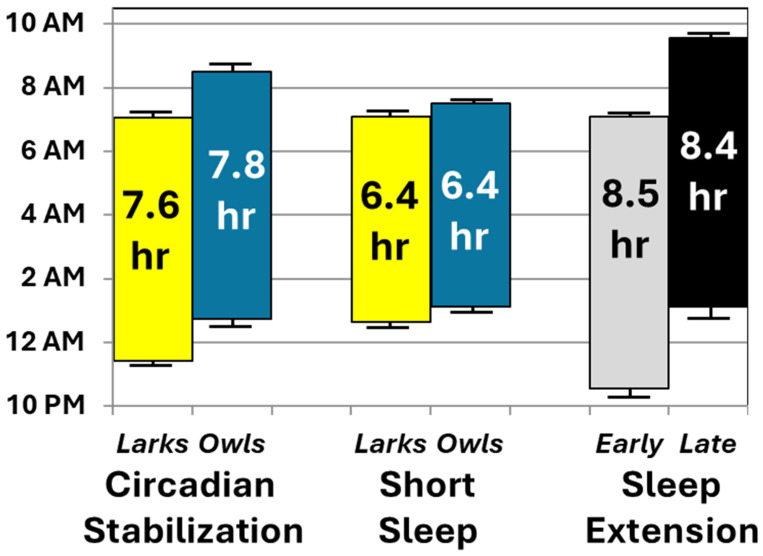
Average sleep periods during key conditions, based on actigraphy. Mean sleep onset and offset are shown at the bottom and top of each bar, respectively, along with their standard errors of the mean. Average length of each sleep period is presented within the bars. For ease of interpretation, data are collapsed by chronotype or randomization except when one of these variables interacted with study condition. When such interactions occurred, chronotype or randomization groups are plotted separately during the conditions in which they showed significant effects in follow-up analyses.

Second, participants’ actual sleep duration and timing, measured via objective actigraphy, conformed well to protocol instructions. Figure 4 summarizes the changes in average sleep periods across the three conditions of primary interest: initial circadian stabilization, sleep restriction, and sleep extension (randomly assigned to early-to-bed vs. late-to-rise arms). To ease interpretation, sleep periods are plotted separately by chronotype and randomized arm only when relevant interactions and significant follow-up tests were found. Actual sleep (Figure 4) closely matched prescribed sleep opportunities in the relevant portions of Figure 1. Sleep duration varied as expected, with both sleep extension conditions averaging within the 8–10-h nightly sleep recommendations [40,41]—approximately 2 h more than during sleep restriction. There was no evidence that these effects on sleep duration varied by chronotype or random assignment to an early vs. late arm for the extension condition. Instead, as intended, chronotype and study arm affected sleep timing, not duration. Chronotype affected timing only pre-randomization (with Larks keeping earlier schedules than Owls), while random assignment affected timing only post-randomization (with late-to-bed resulting in later sleep than early-to-rise). Collectively, these findings support our long-term goal of simultaneously testing the effects of both sleep duration (at realistic levels of short and healthy duration) and timing on health outcomes.

The protocol’s utility was further supported by exploratory analyses of sleep quality. As found in prior adolescent sleep manipulation studies, the shorter the induced sleep period, the higher the proportion of that sleep period was spent asleep, likely reflecting a homeostatic response [11]. Even so, sleep efficiency was high across conditions (87–90%) and the slight differences within the normal range are of questionable clinical significance. Combined with a lack of main or interaction effects of chronotype or random assignment, this lends confidence that the experimental induction of aligned versus misaligned sleep can be performed without differentially inducing confounders in sleep quality or quantity.

The findings also supported the protocol’s utility when examining circadian phase. As intended, there were quite different average circadian phases across chronotypes. At the initial office visit, DLMO averaged 1½ h earlier for Larks than Owls. This effect was not moderated by sleep condition or randomization. These results highlight the protocol’s success in identifying and reinforcing clear chronotype groups, which is essential for inducing aligned versus misaligned sleep periods by adjusting sleep schedules.

Finally, as intended, random assignment to a misaligned sleep extension schedule (late-to-rise for Larks, early-to-bed for Owls) resulted in a misalignment between sleep timing and circadian phase. Compared to the initial circadian stabilization period, phase angle during sleep extension shifted much more dramatically for adolescents randomized to the misaligned arm than those randomized to the aligned arm. For adolescents assigned to a misaligned schedule, their sleep timing during sleep extension was unrelated to their DLMO-measured circadian phase. In contrast, those assigned to an aligned schedule had a strong relationship between their proximal DLMO and midsleep, similar to what was observed in the full sample during the initial circadian stabilization period. Prior publications show that midsleep and DLMO correlate significantly and positively when adolescents can sleep on a relatively unconstrained schedule [25]. The current protocol will allow us to experimentally examine what happens when schedule constraints disrupt that alignment, even in the presence of reasonably healthy sleep duration and quality.

### 3.2. Challenges and Limitations for Future Investigators to Consider

Despite these promising findings, we have encountered some challenges with the protocol that will be important for investigators to consider. Though chosen to maximize enrollment and ecological validity, a sleep-at-home protocol lacks the degree of environmental control of an inpatient setting [42]. Adherence to instructions varies, which can reduce effect sizes. The overall sleep and DLMO patterns we observed here are reassuring, but there are individual differences in how much sleep each adolescent received within each condition, and how aligned or misaligned the sleep extension condition was for them. We advise future researchers to (a) maximize adherence using best practices [23,42]; (b) check for associations between primary outcomes and individual differences in sleep gains across conditions, covarying as needed; and (c) consider using a complementary analytic approach that first examines the experimental effect (aligned vs. misaligned) and then tests whether this effect is statistically mediated by individual differences in degree of misalignment (e.g., the misalignment index calculated here).

Another challenge arose from balancing the practical constraints of family schedules (office visits at 1-week intervals, allowing for three consecutive Friday or Saturday evening sessions) against the scientific goal of promoting distinct chronotypes prior to the sleep restriction and extension conditions. As shown in Figure 1, this involved integrating 4–6-night periods just before the restriction and extension conditions, during which sleep schedules systematically differed across chronotypes by an average of 1½ h. Reassuringly, current analyses suggest we met that scientific goal, but integrating this with weekly office visits meant that the second office visit was not immediately adjacent to a condition of interest. To gather data for each condition, investigators may need to collect primary outcome variables remotely, rather than collecting data exclusively during office visits.

The integration of DLMO assessments into the office visits, while necessary to test whether the protocol was working properly, also raises complications. It has required us to keep participants awake and comfortable in very specific low-lighting conditions [43], while also keeping them sufficiently entertained to return for subsequent visits. Also, DLMO requires data collection well past typical sleep onset [43], which affects that night’s sleep timing and duration. The current protocol leverages this to “jump start” the sleep restriction condition and to maintain chronotype-linked sleep timing differences between the sleep restriction and extension conditions. However, some outcomes of interest could be impacted by distortions in sleep schedules due to DLMO assessment.

Finally, because DLMO assessments necessitated visits that ended late (01:00 for Larks, 02:30 for Owls), arranging participant pick-up was challenging for some families. So far, this has not resulted in differential attrition by demographics or chronotype. However, it could impact initial enrollment in ways that are harder to detect, since families may have differentially opted out after hearing about the early-morning pickup. Although more convenient methods for determining circadian phase are being developed [44,45,46,47], these lack external validation against DLMO in pediatric populations. For now, it will be important for researchers to consider other ways to reduce the burden and promote diversity in experimental sleep manipulation work, as differential enrollment and attrition can distort interpretation [24].

### 3.3. Conclusions

Despite these complications, the present findings are encouraging, suggesting that a sleep-at-home protocol can experimentally test the importance of *when* adolescents sleep, not just *how long*. This is particularly relevant for adolescents, who are developmentally prone to sleep that is both inadequate duration and poorly timed [1,2]. Although the current findings do not immediately inform clinical practice or health policy, we nevertheless consider this work to be an important step in a field that previously lacked relevant, tested experimental methods. The field now has a validated and novel research tool to examine the causal impacts of sleep timing on adolescent health outcomes. The next steps are to use this tool—to apply this protocol—to address applied questions. On a practical level, is sleep extension best conducted via approaches that emphasize early-to-bed (e.g., sleep hygiene), late-to-rise (e.g., delay school start times), or a more individualized circadian-informed strategy? We look forward to using this protocol to help find out.

## 4. Materials and Methods

This research was overseen by the Cincinnati Children’s Institutional Review Board. All parents provided informed consent, and all adolescent participants provided informed assent. Data collection occurred during the summer months to avoid ethical concerns around the educational impact of induced sleep restriction [10,12,13] and because the late-to-rise sleep extension condition was incompatible with common school start times [4,48]. Data reported here were from the first three summers of an ongoing larger project that is examining the impact of changes in sleep timing and duration on dietary intake, mood, and behavior. The deidentified data relevant to this paper are available in the online supplementary materials. This protocol is registered with ClinicalTrials.gov (NCT04992611).

### 4.1. Participants

Participants were recruited via print and electronic ads within and outside of Cincinnati Children’s network, print ads posted at community organizations, and social media advertisements. All ads directed interested parties to an online pre-screener, with a phone number for those who preferred verbal screening. The pre-screener began with a brief description of the study. Prospective participants who remained interested then answered eligibility screening questions, with only those who passed receiving a final set of questions. That final set included family contact information and the adolescent’s typical sleep onset and offset on nights when there were no school or work obligations, modeled after the Munich ChronoType Questionnaire [49]. The middle of the sleep period between onset and offset (“midsleep”) on such nights has been shown to correlate with objective metrics of circadian phase in both adults [50,51,52] and adolescents [25,53]. This approach was selected over measures that ask one’s sense of the “best time of day” for activities because those either have not been validated against a biological circadian marker or show much weaker correlations [54].

Prospective participants whose midsleep on non-school, non-work nights was reported to be at or before 04:00 were identified as potential “Larks”, while those whose midsleep on those nights was reported to be at or after 05:30 were identified as potential “Owls”. These two groups were then targeted for more detailed telephone follow-up; the “intermediate” group between them was thanked for their interest but not pursued further. The telephone follow-up for prospective Larks and Owls included more details about the study demands, as well as formal screening for exclusion criteria.

All adolescents were required to be 14.0–18.9 years old at the time of study participation and to fit into the Lark or Owl chronotype as described above. Exclusion criteria included the use of medication known to affect sleep, melatonin levels, or diet; intellectual disability; symptoms of insomnia, obstructive sleep apnea, or periodic limb movement disorder [55,56,57,58,59,60]; obligations that interfered with study sleep schedules; and daily intake of >1 coffee or energy drink, or >2 caffeinated sodas. This threshold was set to allow common caffeine intake while avoiding potential artifacts from heavy caffeine use or withdrawal [61]. Related to requirements of the ongoing study, we excluded body mass index > 30. For safety, we also excluded participants with neurological conditions; those who refused to refrain from automobile driving during sleep restriction; and depression, psychosis, or bipolar disorder based on a structured diagnostic interview [62].

### 4.2. Experimental Protocol

Figure 1 provides a graphical overview of the 3-week protocol involving 3 office visits. Throughout the study, all adolescents slept at home, monitored by wrist-mounted actigraphs (see below). A sleep-at-home design was selected over an inpatient admission out of concern that only highly selected adolescents/families would complete a protracted inpatient study. Participants received compensation at each office visit (USD 100 for Visit 1, USD 150 for Visit 2, and USD 200 for Visit 3), regardless of adherence to sleep instructions. However, participants who were markedly non-adherent during an office visit (e.g., failure to wear the actigraph or sleep–wake schedules differing by more than 2 h from instructions) were not allowed to continue. Participants were also eligible for up to USD 60 for completion of measures that were part of the larger study, but not the focus here.

#### 4.2.1. Circadian Stabilization (SBN; Nights 1–6)

The protocol opens with a 6-night chronotype-aligned SBN condition, designed to reinforce distinct circadian timing across chronotypes and to homogenize sleep duration prior to sleep restriction. To promote adherence and retention, adolescents were allowed some control over their SBN schedules. Larks were asked to select an 8-h period with bedtimes ranging from 21:30 to 23:00; Owls selected an 8-h period with bedtimes ranging from 00:30 to 02:00. Instructions for maintaining that schedule and how to use the actigraphs were reviewed via telephone and reinforced in writing as part of a mailed shipment to families prior to starting the study.

At the end of the initial SBN, adolescent participants and their parents attended an office visit, beginning at 18:00 for Larks and 19:30 for Owls to accommodate the DLMO assessment (see below). During the first portion of the visit, participants rotated through several activities: completing background questionnaires, reviewing actigraphy data from the previous week and instructions for the following week’s sleep, and completing measures related to the larger project. Parents then left, returning to pick up the adolescent after a formal DLMO assessment.

#### 4.2.2. Sleep Restriction (SR; Nights 7–11)

During this 5-night condition, adolescent sleep opportunity was restricted to 6½ h/night, in line with our prior work and with a common level of sleep restriction for adolescents on school nights [9,11,12,13,14,15,16,18,21,24,63,64,65]. The first night of SR immediately followed the first office visit, which ended late (01:00 for Larks and 02:30 for Owls) to accommodate DLMO assessment. Accounting for transit time, the rise time on the first morning of SR was set to 08:00 for Larks and 09:30 for Owls. Across the subsequent 4 nights, bed and rise times were the same for both chronotypes, 00:30–07:00, falling between the two SBN schedules and mimicking a plausible SR period on school nights.

The SR condition was then followed by 2 nights back on the SBN schedule. Past research indicates that 2 nights on a chronotype-preferred schedule can allow for a significant “bounce back” of circadian timing after 5 nights on a school-night sleep schedule [66]. Adolescents and their parents then returned for a second office visit. That visit included uploading and review of actigraphy data from the previous week, instructions for the following week’s sleep schedule, and DLMO reassessment. The subsequent morning, they were again allowed to awaken later to accommodate the DLMO assessment, followed by another night on the SBN schedule. Collectively, this resulted in 4 consecutive nights following the SR condition, during which Larks were placed on a sleep schedule approximately 1½ h earlier than Owls.

#### 4.2.3. Sleep Extension (EXT; Nights 16–20)

Participants closed the study with a 5-night period of sleep extension, comprising 9½ h of sleep opportunity, which has been shown to allow for consensus recommendations for nightly sleep duration [9,11,12,13,14,15,16,18,21,24,64,65]. Importantly, the timing of EXT was randomly assigned to an early-to-bed arm (21:30–07:00) versus a late-to-rise arm (00:30–10:00). The temporal separation between these arms was intentionally set, with the midpoint of sleep opportunity for each arm being 1¾ h apart from the midpoint of SR, similar to average adolescent weeknight–weekend shifts [63]. The goal of random assignment was to experimentally induce EXT arms that were either aligned with adolescents’ circadian phase (early-to-bed for Larks, late-to-rise for Owls) or misaligned (early-to-bed for Owls, late-to-rise for Larks). After the 5 nights of EXT, participants and their parents returned for a final office visit, which included a review of accelerometry data and DLMO assessment.

### 4.3. Delivery of Sleep Instructions

Instructions for sleep schedules were provided by phone prior to starting the SBN condition, in person at the first two office visits, and in writing throughout. As described in our prior papers, each verbal conversation was individualized and used established principles of pediatric psychology and behavioral sleep medicine: (a) pre-planning, (b) conjoint identification of barriers and problem-solving with parent and adolescent, (c) development of positive bed/rise routines, (d) activity planning and commitment, (e) self-monitoring with regular objective feedback, (f) frequent contacts using electronic check-ins and prompts, and (g) positive reinforcement [11,23]. Although the discussion focused on the adolescent to respect their emerging autonomy, parents were present to offer support, remove obstacles, and provide in vivo reinforcement at home [42].

To maximize engagement, minor adherence slips were addressed non-judgmentally as challenges and opportunities for problem-solving [42]. Participants were asked not to nap, to avoid caffeine on the day of an office visit, and to have no more than 2 caffeinated sodas, or 1 coffee or energy drink, on other days of the study. Adolescents were asked to give a good faith effort to conform to sleep instructions and reassured that, while they may not fall asleep immediately after an early bedtime or sleep until a late rise time, we have observed youth to extend their sleep further than they expected.

Each conversation was unique, but there were some common elements, especially when study schedules differed markedly from an adolescent’s usual sleep. To facilitate unusually late bedtimes, participants were encouraged to brainstorm engaging activities that prevented sleep onset (e.g., avoiding lying down). Similarly, unusually early rise times often prompted discussion of alarms, family support, and pre-planned activities that are incompatible with falling back asleep. For unusually early bedtimes, important activities were scheduled earlier, a brief calming routine was established, and prompts ensured timely “lights out”, with electronic devices turned off or silenced. For unusually late rise times, provisions often were put into place to keep the bedroom dark/quiet and adolescents were encouraged to give themselves liberal opportunity to fall back asleep. Conversations focused on bedtimes and rise times, rather than light exposure per se, but it was not uncommon to discuss dim lighting prior to bedtime, darkness during the sleep period, and turning on lights when awake for the day. During discussions, staff added brief notes/prompts on the written instructions for participants, and families were encouraged to call with any questions.

### 4.4. Measures

#### 4.4.1. Sleep Behaviors

Throughout the study, adolescents were asked to wear wrist-mounted actigraphs (Ambulatory Monitoring Inc. [AMI], Ardsley, NY, USA) nightly and to complete a daily sleep diary that included questions about the prior night’s sleep onset and offset. As in our past work, actigraphy data were uploaded at each office visit and reviewed with adolescents and parents [9,11,12,14,15,17,18,22,23,64,65,67,68], comparing data against the sleep diary to cue discussion about adherence and to screen for artifacts. The AMI scoring algorithm has >90% agreement with EEG-defined sleep [69]. This allowed us to derive an objective nightly estimate of sleep onset, offset, sleep period duration (onset to offset), midsleep (midpoint of the sleep period), and quality (sleep efficiency; % of sleep period spent asleep). Data were averaged across nights within each of three key study conditions: initial SBN, the four nights of SR with 00:30–7:00 schedules, and EXT. Interpretable data were available on >90% of nights within each condition. As a quality check, exploratory analyses excluded the 6% of conditions for which data were available for less than half the requested nights; these yielded the same findings as those reported above.

#### 4.4.2. Circadian Phase

Circadian timing was determined during each office visit based on salivary dim-light melatonin onset (DLMO), the best-established circadian phase marker in humans [70,71]. DLMO is assessed via serial saliva sampling to detect the point at which melatonin levels inflect from the low levels common during wakefulness to a rapid ascent in preparation for sleep onset [43]. To maximize the chances of capturing that inflection, the office visits for Larks ran from 18:00 to 01:00, and those for Owls ran from 19:30 to 02:30. During that entire period, participants were awake but in dim light (<5 lux), supervised by study staff. When not meeting for sleep reviews, participants were in a common area, where they could stream or view age-appropriate content on dimmed screens, play games, or complete study measures. Bathroom breaks, also in dim lighting, were allowed except in the 10 min before each sample to avoid potential postural or activity artifacts. Samples were collected every 30 min using Salivettes, centrifuged and frozen on-site, and shipped on dry ice to SolidPhase, Inc. (Portland, ME, USA), for direct radioimmunoassay. DLMO was computed as the time melatonin values surpassed 4 pg/mL [72,73]. Out of 202 nights with complete melatonin sampling, 10 (<5%) did not show a clear crossing of that threshold. When all values remained below 4, DLMO was set at ½ hour after the final sample; when all values were above 4, DLMO was set at ½ hour before the first sample. As a quality check, exploratory analyses excluded those 10 nights, with no change in findings.

#### 4.4.3. Circadian Misalignment

A misalignment index quantified the change in the relationship between circadian phase (based on DLMO) and sleep timing (based on actigraphy) from SBN to EXT. As an initial step, we calculated phase angles for each condition. For SBN, phase angle was defined as the arithmetic difference, in hours, between midsleep during SBN and DLMO during the first office visit. For EXT, this was the difference between midsleep during EXT and DLMO at Visit 2. For each participant, the misalignment index reflects the absolute value of the difference between these two phase angle values. Higher misalignment index scores indicate greater deviation during EXT than would be expected based on initial SBN.

#### 4.4.4. Other Measures

Demographic characteristics, including adolescent age, sex, and race/ethnicity, were collected via parent-reported background questionnaires during the initial office visit. As part of the larger ongoing project from which the current data were drawn, other measures assessed dietary intake, preferences, mood, and behavior, though these are not included in the current analyses.

### 4.5. Analytic Approach

Sample characteristics are described using means ± standard deviations for continuous variables and percentages for categorical variables. Between-subjects *t*-tests or chi-square tested for differential attrition (Success Metric 1), comparing those who completed the full study against those who started but did not complete the study. Analyses for the other success metrics focused on sleep within the three conditions noted above—initial SBN, SR, and EXT—and DLMO at the three office visits. To assess Metric 2 (intended changes in sleep duration), repeated-measures linear models compared sleep period duration across the within-subjects variable of study condition and the between-subjects variables of chronotype and randomly assigned EXT arm (early-to-bed vs. late-to-rise). To assess Metrics 3 and 4 (DLMO difference across chronotypes, the effect of the randomization on sleep timing), these analyses were repeated with DLMO and midsleep as the outcome variables, respectively. Follow-up analyses for significant interactions involving the within-subjects variable of condition were probed with *t*-tests within each condition. Main effects in the absence of interactions were probed with *t*-tests (between subjects) or Tukey’s Least Significant Difference (within subjects). Finally, Metric 5 was assessed in two ways: (a) Spearman correlations were calculated between midsleep and DLMO during the office visits proximal to initial SBN and EXT, and (b) linear models tested the differences across the aligned versus misaligned EXT conditions on the misalignment index, checking for moderation effects of chronotype and EXT arm (early or late) in separate analyses. Balancing the risk of Type 1 and 2 errors given the number of analyses and sample size, we applied a 2-tailed significance threshold of 0.01.

## Figures and Tables

**Table 1:** Glossary of key terms and acronyms.

Term	Definition
Circadian rhythms	Oscillations of physiological processes across a 24 h period, sometimes called a “body clock”. For example, in a healthy individual, core body temperature peaks in the late afternoon to evening and dips to a trough in the early morning hours.
Circadian phase	The timing of a circadian rhythm (body clock). People differ in the time of day associated with that rhythm—some earlier, some later.
DLMO (dim-light melatonin onset)	A common circadian phase biomarker. In a healthy person, melatonin levels in serum, urine, or saliva are low during most wakefulness and elevated during sleep. DLMO indexes the timing of the abrupt rise, which normally occurs a few hours before habitual bedtime.
Chronotype	An individual’s timing of sleep onset, offset, and other behaviors when not constrained by social schedules. For example, “Morning Larks” (or simply “Larks”) prefer early bedtimes and rise times, and “Night Owls” (“Owls”) prefer late bedtimes and rise times. “Intermediate” types fall in between. Chronotype is expressed relative to a distribution at a given age; bedtimes and rise times normatively shift across the lifespan, but at any given age there is still a distribution of Larks, Owls, and Intermediate types. This study enrolled adolescent Larks and Owls based on their reported sleep schedules when they did not have external demands.
Midsleep	The midpoint of the period between sleep onset and offset. For example, if sleep onset is midnight (00:00) and offset is 08:00, midsleep is 04:00. For this study, midsleep was used (a) at the time of enrollment as a measure of chronotype and (b) during the protocol as a measure of sleep timing during a sleep manipulation condition.
SR (sleep restriction)	Sleep duration that is shorter than has been recommended by experts (e.g., less than the recommended 8–10 h/night for adolescents). For this study, the SR condition was induced by setting bedtimes and rise times 6.5 h apart.
HS (healthy sleep)	Sleep duration that meets expert recommendations. For this study, the HS condition was induced by setting bedtimes and rise times 9.5 h apart, randomized to two arms: (a) HS that was timed to be relatively aligned with circadian phase (see below) vs. (2) misaligned.
SBN (phase stabilization)	A run-in condition of 8 h/night in bed that was designed to reinforce the distinct circadian timing of Larks versus Owls, and to homogenize sleep duration prior to the SR condition.
Circadian alignment	The degree to which an individual’s behaviors occur at a time that temporally aligns with their circadian phase.
Circadian misalignment	The degree to which an individual’s behaviors occur at a time that differs from their circadian phase. Misalignment can happen because of personal choices or external demands. For example, early school start times place an external demand on sleep schedules, forcing many adolescents with late circadian phases to awaken much earlier than fits their body clocks. For this study, half of the youth were randomized to an HS condition that was timed to be relatively aligned with their circadian phase, and half were randomized to an HS condition that was misaligned with their phase.
Phase angle	A metric that compares behavioral sleep timing with a circadian biomarker. For this study, phase angle was defined as the time interval between DLMO and midsleep.
Misalignment index	The absolute value of the difference in phase angle between HS and SBN. Higher scores indicate an HS condition that is relatively misaligned with circadian phase.

**Table 2:** Characteristics of participants who initially enrolled and completed the study.

	Enrolled	Completed	*p*-Value
Sample size (n)	81	59	
Age (Mean ± SD in years)	16.3 ± 1.4	15.8 ± 1.2	>0.10
% Female	51.9	52.5	>0.10
Race/Ethnicity			>0.10
% Non-Hispanic White	64.2	69.5	
% Non-Hispanic Black	24.7	16.9	
% Non-Hispanic Multi-Racial	3.7	3.4	
% Hispanic White	4.9	6.8	
% Other or Not Reported	2.5	3.4	
% Lark Chronotype	49.4	52.5	>0.10

Note: *p*-values are based on a 2-sample *t*-test or chi-square, reflecting a comparison of those who completed the study against those who initially enrolled but did not complete it.

**Table 3:** Summary of linear model effects.

Outcome Variable	Effect Sizes (Partial Eta-Squared) for Each Predictor and Interaction					
	Condition/Visit	Chronotype	Random EXT Arm	Condition X Chronotype	Condition X EXT Arm	3-Way Interaction
Sleep Period Duration	0.886 **	0.004	0.000	0.012	0.007	0.127
Midsleep	0.292 **	0.593 **	0.335 **	0.727 **	0.891 **	0.017
DLMO	0.200 *	0.207 *	0.000	0.142	0.296 **	0.039

* *p* < 0.01; ** *p* < 0.001. Analyses tested the main effects of the within-subjects effect of sleep condition (SBN, SR, EXT), the between-subjects effects of chronotype (Lark, Owl) and randomized EXT arm (early-to-bed, late-to-rise), and within X between-subjects interaction effects. The text details significant findings, including follow-up tests of significant interactions.

## Data Availability

The original contributions presented in this study are included in the supplementary material. Further inquiries can be directed to the corresponding author.

## Supplementary Materials

The following supporting information can be downloaded at: https://www.mdpi.com/article/10.3390/clockssleep7010004/s1, raw data for reported variables.
